# Supplementary figures and images for: Probabilistic, entropy-maximizing control of large-scale neural synchronization
Source: PLoS One. 2021 Apr 30;16(4):e0249317. doi: 10.1371/journal.pone.0249317 (PMC8087389; doi:10.1371/journal.pone.0249317)

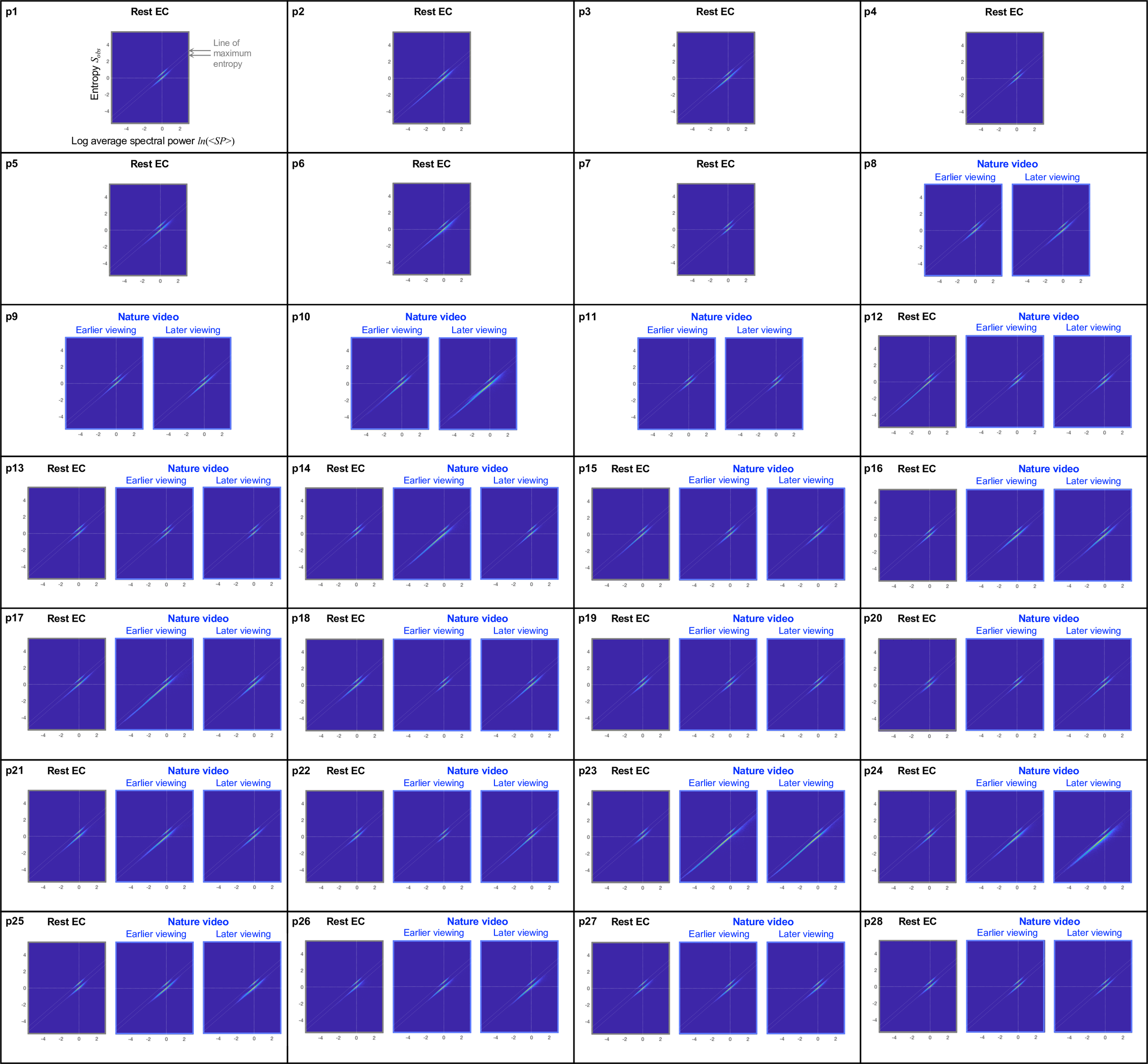

Supplement: S1 Fig — All participants who participated in the nature-video condition provided data for both earlier and later viewings. The dynamic ranges of average spectral power (for d = 3 sec intervals) were moderately to substantially extended along the line of maximum entropy (the gray dashed oblique lines) for the actual EEG data relative to their phase-scrambled controls for all participants for all conditions. Note that the degree of extension of spectral-power dynamic range does not appear to be a trait-like property as it substantially varied across conditions (even between the two instances of the nature-video condition) for some participants. (TIF) [file pone.0249317.s001.tif]

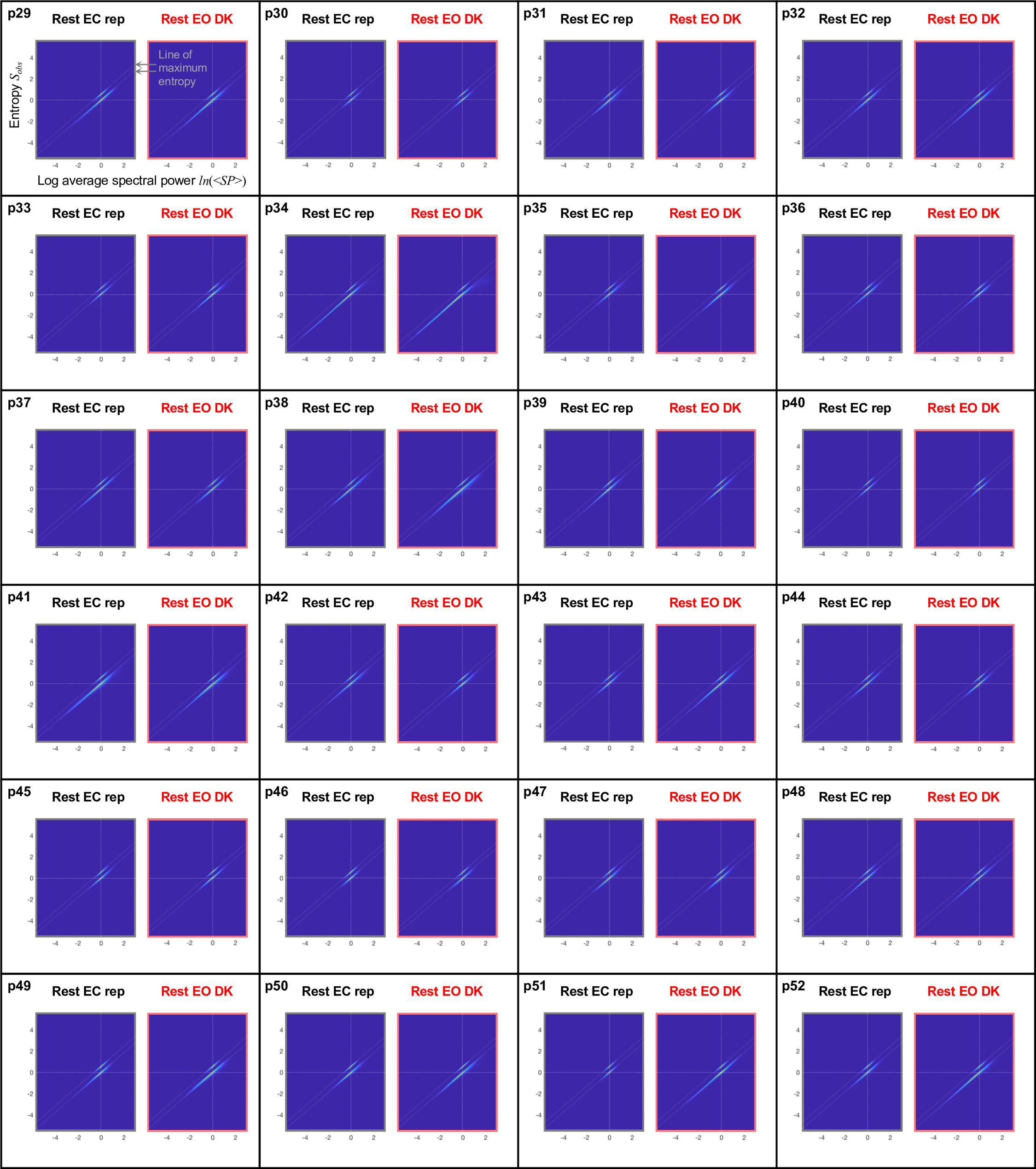

Supplement: S2 Fig — The dynamic ranges of average spectral power (for d = 3 sec intervals) were moderately to substantially extended along the line of maximum entropy (the gray dashed oblique lines) for the actual EEG data relative to their phase-scrambled controls for all participants for all conditions. Note that the degree of extension of spectral-power dynamic range does not appear to be a trait-like property as it substantially differed between the two similar conditions for some participants. (TIF) [file pone.0249317.s002.tif]
