# Supplementary material for: Probabilistic, entropy-maximizing control of large-scale neural synchronization
Source: PLoS One. 2021 Apr 30;16(4):e0249317. doi: 10.1371/journal.pone.0249317 (PMC8087389; doi:10.1371/journal.pone.0249317)
Supplement: Attachment — Submitted filename: MencelogluEtAl_pone_R1_response.pdf [file pone.0249317.s003.pdf]

Our point-by-point response to the two reviewers' thoughtful comments.
The original review is reproduced in black text and our responses are highlighted in blue text.

Reviewer #1: In this paper, the authors used a simple chain model of spreading synchronization to account for deviation from purely stochastic dynamics in spontaneous EEG under various experimental conditions. The link between the model and the EEG data was constructed by the assumption that the size of a synchronized region is proportional to the observed spectral EEG power. This is a reasonable assumption.

They showed that (1) spectral power distribution of real EEG deviates from its stochastic (phase scrambled) counterpart in a u shaped manner, elevating high and low powers, (2) both stochastic and real EEG obey the maximum entropy relationship predicted by the model for a constant probability of spreading termination, (3) for longer time windows above 3 seconds, however, there was a deviation, indicating that the deviation of real EEG in the low and high power ranges are associated with relatively slow mechanisms, and (4) that these deviations from stochastic behavior consistently featured particular spatial patterns across brain regions (only rough, because surface Laplacian was used). The authors offer some (fairly speculative) explanation for the meaning of these findings.

This study is carefully conducted and yielded interesting and (in my view) highly relevant results that could also help to build and understand more biologically realistic models of neural dynamics. In addition, the presented methodology may be very useful for further research. As far as I can judge, all theory is consistent and well founded. The paper is very well written and instructive. I enjoyed reading it.

As a conclusion, I am inclined to recommend unconditional acceptance (which I very rarely do).

We thank the reviewer for carefully reading the manuscript, and we appreciate his generous comments. In particular, we could not think of a greater compliment than "I enjoyed reading it."

Nonetheless, I have a few suggestions for the authors:
1) In the introduction, especially in the first paragraph, it sounds as if brain dynamics is exhaustively described by interacting (harmonic) oscillations. What about transient dynamics and oscillations that are not at all sinusoidal? Detailed neural and neural population models predict this. Does the Fourier decomposition not generate a biased representation here? Please comment.

We appreciate these comments. We now acknowledge the limitations of our results due to the use of a time-frequency decomposition approach in the discussion section.

At the end of the fourth last paragraph, we added, "Further, non-oscillatory neural activities obscured in frequency-decomposition approaches also likely play a substantial role in precisely timed neural coordination."

We also added a new paragraph just above the concluding paragraph. "While the current analyses used a time-frequency decomposition approach that extracted sinusoidal components from EEG, neuronal oscillations are not necessarily sinusoidal (though oscillatory activities of large neural populations, as reflected in EEG, tend to approximate sinusoidal waveforms due to spatial averaging [48]). Non-sinusoidal waveforms are detected by time-frequency decomposition methods as the sinusoidal components with the approximately matching frequencies as well as additional sinusoidal components at various harmonic frequencies that may spuriously mimic phase-to-amplitude coupling [e.g.,49,50]. We observed entropy-maximizing behaviors on the timescale of a few seconds for sinusoidal components of a broad range of frequencies. Thus, even if some of the observed sinusoidal components reflected the fundamental and harmonic components of non-sinusoidal oscillations, we could still infer that the underlying non-sinusoidal oscillations exhibit entropy-maximizing behaviors on the timescale of a few seconds in the sense that their sinusoidal components do."

2) Page 13, last para: typo “2D-density”

Fixed.

3) Fig. 5A: please indicate the scaling of the x axes.

We thank the reviewer for catching this. The x-axis label got hidden while we futzed with the figure panels. The x-axis scale is normalized to the standard deviation of the distribution for the phase-scrambled controls. The x-axis labels are now visible.

Reviewer #2: This manuscript is about the mechanisms underlying the large scale synchronization of the brain activity. This is an important and interesting issue that deserves a careful analysis with well defined and documented hypotheses. The reviewer does not reject the overall approach proposed by the authors but rather invite them to revise in depth the rationale of the work. There is a huge confusion between the a priori model, the 'thermodynamic' point of view (entropy, energy, adiabatic transform) and the results. There is too much vague statements and the mathematics, although very simple in terms of the developments, deserve some justifications.

I details, the comments are the following (they aimed at helping the resubmission of this work)

We thank the reviewer for carefully reading the manuscript to provide numerous comments. We also appreciate that the reviewer intends their comments to be constructive.

- Introduction: the authors should explain how the 'spectral power dynamics' (the original one and the phase-resampled ones) can help to explain synchronization dynamics. Of course, phase scrambling is a way to destroy coherence between harmonic component (distortion) but a simulation would be convincing.

We assume that spectral power is proportional to (or at least monotonically related to) the number of neurons engaged in synchronous oscillatory activity. To our knowledge, this assumption is broadly accepted in the literature. Nevertheless, we clearly state in the paper that we make this assumption.

We have difficulty understanding what types of “simulations” the reviewer is referring to and what they mean by “convincing.” Phase scrambling randomizes the cross-frequency phase relations that generate the specific temporal sequence of spectral power in the actual EEG data while preserving the time-averaged spectral power. Thus, it is reasonable to assume that non-stochastic aspects (i.e., temporal structures) of actual EEG spectral-power dynamics can be revealed by comparing spectral-power time series between the actual and phase-scrambled versions of EEG data.

The reviewer did not understand how 'the phase-scrambled spectral-power dynamics are characterized by exponential power distributions': distribution over what?

We apologize for the imprecise statement. It refers to spectral-power distribution over time. We have revised the relevant section as follows: “Because stochastic dynamics reflect a Poisson process, phase-scrambled spectral-power dynamics are characterized by an exponential temporal distribution of power.”

The dynamics raises the issue of stationarity of the recordings. How much such a distribution is reliable?

We agree that "stationarity" is often a concern for EEG analyses. However, we are not entirely clear on what the reviewer means by “How such a distribution is reliable.” As we understand, EEG stationarity can be evaluated with respect to any temporal statistic (e.g., temporal average, temporal variability, etc.). EEG stationarity with respect to different temporal statistics may reflect changes in different aspects of macroscopic neural states. Our results have shown that spectral-power dynamics reflect a stationary Poisson process (inferred from the entropy-maximizing behavior of spectral-power dynamics) up to the timescale of a few seconds.

The authors introduce the 'probability of synchronization' and the principle of 'maxEnt' together with the 'energy efficiency'. All those notions should be carefully defined in the present context.

These concepts are discussed in the Results section. We agree that main concepts are typically discussed in the introduction section. However, for this paper, we feel that it is more effective to elaborate on the probability of synchronization, entropy maximizing dynamics, etc., in parallel with developing the mathematical model in the results section. We think that it is helpful to explain the relevant concepts while precisely defining them as model parameters. We also believe that the development of both the concepts and model naturally follow the presentation of Figure 2 that demonstrates the characteristic U-shaped deviations of EEG spectral-power dynamics from exponential, providing the premise of the study. The goal of the current study was to understand the mechanisms that generate these U-shaped deviations. We tried to

alleviate the reviewer's concern by adding "(see Results)" in the relevant part of the introduction section. We were also encouraged by the fact that Reviewer 1 found the general structure of the paper to be effective.

Finally, this section should give the hypotheses underlying the objectives of the work.

We agree that we provide only a brief description of the hypotheses in the introduction section, in terms of two general mechanisms that might generate the U-shaped deviations of EEG spectral-power dynamics from stochastic dynamics. Nevertheless, as we explained above, we believe that the mechanistic aspects of our hypotheses are more effectively elaborated in parallel with the development of the mathematical model in the results section.

- Methods:
To be able to reproduce the results, some of the methodological steps deserve more justification.

1) What was the criteria to select the ICA component(s) to be removed?

The ICA component(s) reflecting blinks were visually identified and removed. Although the process may seem subjective, blink-related components typically have a distinct scalp distribution (focused on mid-anterior electrodes that reflect eye movements) and are among the largest components (often top 10%, if not the first or second component). Therefore, it is straightforward to visually identify the blink-related component(s) by inspecting the scalp maps of all components. To validate the blink-related component(s), we compared the time courses of the blink-related component(s) and the raw EEG to confirm that the blinks that were reflected in the raw EEG and in the component(s) temporally coincided. We also made sure that removing the specific component(s) removed blink-related activity without distorting the EEG waveforms by overlaying EEG traces from before and after the blink component removal; EEGLAB's runica function (Makeig, Bell, Jung, & Sejnowski, 1996) facilitates this comparison. We have augmented the relevant section of the methods section, but we do not present all these details because the procedure is standard and well documented in EEGLAB.

2) Please specify the parameters of the Current Source Density filtering of the EEG. This is an interesting and important pre-processing. What is the impact on the results? The authors should stress that without solving explicitly an inverse problem to map the EEG onto the cortical substrate, they tend to describe the underlying source activities. This is a good idea that should be more emphasized.

The "smoothness" value we used for the Perrin algorithm was $\lambda = 10^{-5}$ (recommended for 64 channels; Cohen, 2014). This information is now included in the methods section. Surface-Laplacian transform is essentially spatial high-pass filtering so that it would not generate non-stochastic dynamics out of stochastic raw EEG or render non-stochastic EEG stochastic. If there were non-stochastic signals equivalently affecting all electrodes, they would be removed by the transform, but such signals would likely reflect artifact. Based on our experience, surface-

Laplacian transform substantially improves EEG spatial resolution (relevant to Figure 6). We routinely apply the transform to EEG data as there is sufficient evidence (to our knowledge) that it provides an effective data-driven method to infer cortical source activities. Based on our experience, applying the transform also narrowly confines ocular and facial muscle artifacts, virtually eliminates reference-electrode dependence, and substantially reduces volume conduction effects (fairly independent dynamics can be identified in adjacent sites in a 64-channel montage). As recommended by the reviewer, we now articulate these advantages of applying surface-Laplacian transform to EEG data.

3) The EEG temporal derivative is nothing but a high-pass filter. The justification with the 'trigonometric properties' is a bit naive and the (scaling-) exponent beta seems to be neglected although it may be different from 1 and even changing with time. Why does the authors do not use the next coming wavelet framework to 'liter out' this scaling property?

We thank the reviewer for raising this point. As stated in the paper, we have verified that all spectral backgrounds were effectively flattened (per participant per condition) for our EEG data, implying $\beta \sim 1$ for our data when averaged over time. However, we agree with the reviewer that β is known to fluctuate over time due to a variety of mechanisms (relevant studies are cited in the manuscript). We have thus substantially toned down our claim about the effectiveness of taking the temporal derivative in highlighting oscillatory activity. Nevertheless, taking the temporal derivative of EEG offers additional advantages of virtually eliminating drifts and providing a macroscopic measure of the neural currents that generate the observed scalp-EEG potentials. These points are articulated in the revision.

We are not familiar with the “next coming wavelet framework” which the reviewer claims to dissociate oscillatory activity from spectral backgrounds. We conducted a fairly extensive literature review on the $1/f^{\beta}$ spectral background, but we did not come across any algorithm that continuously dissociates oscillatory activity from the spectral background. In fact, a recent study that attempted to dissociate α oscillations from the $1/f^{\beta}$ background used the method of subtracting the spectral background by fitting log-log-linear and bell-shaped functions to time-averaged power spectra (Ouyang et al., 2020, Decomposing alpha and 1/f brain activities reveals their differential associations with cognitive processing speed, *NeuroImage*). A similar subtraction technique is also discussed in a recent methodological article (Donoghue et al., 2020, Parameterizing neural power spectra into periodic and aperiodic components, *Nature Neuroscience*). Regardless, it is not crucial for the purpose of the current study to cleanly dissociate oscillatory and background spectral activities. In fact, it is likely that many neurophysiological mechanisms simultaneously influence both the oscillatory and background spectra (slope, overall magnitude, etc.). Our goal was to elucidate the mechanisms that make the temporal distributions of EEG spectral power deviate from stochastic (exponential) distributions in the characteristic U-shaped manner (Figure 2). Our results suggest that the prevalent low-power and high-power states are generated by slowly adjusting (on the timescale of a few seconds) a parameter that can be interpreted as the probability of synchronization (based on our mathematical model) while maintaining maximum entropy. Future studies may

investigate how the mechanisms that influence oscillatory and background spectra may differentially contribute to this process.

4) Spectral power as a function of time. This is a difficult problem that raises, as previously mentioned, the issue of stationnarity. The time-frequency indeed offers a framework to handle this problem but the method is not described neither discussed in terms of the choice of the wavelet itself (a DOG wavelet would have been more appropriate). The author should better describe the method here.

To our knowledge, Morlet wavelets have been commonly used for time-frequency decomposition of EEG data. Nevertheless, we agree with the reviewer that commonly used does not mean appropriate. That said, we do not see any disadvantages of using Morlet wavelets for time-frequency decomposition for our purposes. Morlet wavelets are simple sinusoidal templates with Gaussian envelopes, that effectively dissociate amplitude and phase. They are limited by their temporal and spectral widths, but we clearly indicate the temporal and spectral resolutions of Morlet wavelets that we used so that the reader can judge for themselves the degree to which our conclusions may be limited by the wavelet tunings in the temporal and spectral domains. We have expanded the description of Morlet wavelets in the revision.

The reviewer states that “a DOG wavelet would have been more appropriate.” Our understanding is that “DOG” stands for difference-of-Gaussian. Difference-of-Gaussian wavelets would be appropriate for detecting the time points of abrupt changes in spectral power. In fact, difference-of-Gaussian wavelets are useful for edge detection while analyzing natural visual scenes; the retinal ganglion cells have receptive fields that act like difference-of-Gaussian filters. It is unclear to us how difference-of-Gaussian wavelets would be effective for tracking spectral power over time; for instance, they would yield no signal when spectral power remains high but constant. Regardless, for the reasons stated in the preceding paragraph, we see no reasons why the use of Morlet wavelets would be disadvantageous for our purposes.

5) The entropy introduced in 3.4 is not justified and the material given in the first pages of the results should be placed in the method. Jumping in this part (Results), the authors should stat with the Poisson distribution (Eq.3) to describe the neural population participating to a synchronized process (so giving a significant amplitude somewhere in the power spectrum).

It is unclear to us what the reviewer means by “justifying” the entropy introduced in 3.4 (especially since we are using the standard equation that defines entropy). We do explain how we chose the number of bins necessary to compute entropy from numerical data. We used the formula developed by Freedman and Diaconis (1981) to compute the optimum number of bins as stated in 3.4. The reasons why we computed entropy are provided in the results section. As we stated above, because the premise of the current study is the U-shaped spectral-power distributions (relative to exponential) demonstrated in Figure 2, we believe that it is appropriate to develop the model in the results section in parallel with evaluating the EEG data in relation to the model predictions.

It was not clear for the reviewer why 'the exponential form of [the Poisson density] indicates that the model yields maximum entropy for fluctuations n, for a given temporal average '?

For a distribution of any random variable when there are no constraints, entropy (S) is maximal (i.e., S=log(N) where N is the number of possible values) when the distribution is flat (all values being equally probable, p=1/N). However, for a distribution with a specific average, entropy is maximized when the distribution is exponential. One can prove this by finding the relationship between p_i's and n_i's (the random variable in our case: the number of synchronously oscillating neurons) that maximizes the entropy equation (3.4) with the constraints that (1) p_i's sum to 1 (the total probability has to be 1) and (2) the expectation value of n is a specific number. Lagrange multipliers can be used to maximize entropy given these constraints to yield the exponential relationship between p and n. We now briefly mention this procedure.

The figure2 deserves more explanation about the origin of the horizontal index (n?) and what we understand with 'residual deviation from...' (something to do with the Kullback-L divergence metric?). The exact definition of the defendant variable should be given.

We apologize that this crucial figure (providing the premise for the current study) was unclear. We substantially revised the labels and caption for Figure 2 to clearly articulate the U-shaped deviations of EEG spectral-power probability distributions from exponential.

6) It sounds that the objective of this methodological development is Eq.13. The relationship between the entropy introduced in 3.4 (positive sign because of the discrete nature of the p's ?) and the entropy introduced in Eq.6 is not clear.

We appreciate the reviewer for detecting the missing negative sign in 3.4. This mistake has been corrected. Entropy for the EEG data needs to be computed based on discrete probability distributions (3.4) whereas entropy can be theoretically computed over a continuous probability density function to yield an analytical expression (Eq. 6).

7) The duration 'd' has something to do with an assumption of stationarity. Shouldd be commented.

"d" is a duration over which the entropy and average of spectral-power probability distribution were computed. If the distribution over d is exponential (i.e., if the points defined by the entropy and log average spectral-power computed over different intervals of d fall along the line of maximum entropy defined by the phase-scrambled data), we can infer that the system (in this case the fluctuation of spectral power) maximizes entropy. Based on Eq.1-3, if the probability distribution of spectral power over d is exponential, we can also infer that the quantity p (whose most straightforward interpretation is the probability of synchronization) is stationary over d. Therefore, there is no "assumption of stationarity" regarding d that needs to be justified. Rather, we experimentally determined the maximum duration d over which the quantity p remained stationary.

8) The maximum entropy principle is not clear in the development

There is no “maximum entropy principle” that needs to be explained or justified for this study. We computed entropy for the temporal distributions of EEG spectral power using the standard equation and showed that EEG spectral power fluctuated in such a way that it maintained maximum entropy over the duration of a few seconds. This is our experimental finding. Using a simple mathematical model, we then inferred that the finding is consistent with the interpretation that the macroscopic probability of synchronization is adjusted on a slow timescale of a few sections.

The Results are difficult to appreciate and the Discussion is rather speculative (and it sounds strange to see an hypothesis expressed in this section). The parameter p_term should be better interpreted (and justified). The interpretation as a probability is far from its introduction in (3)
If the main result is about the 'EEG dynamics that closely follow the line of Max Entropy for all representative frequencies', the manuscript is not convincing, neither the figures (3) an (4).

We are a bit confused as to what is not convincing about the data presented in Figures 3 and 4; perhaps the reviewer might elaborate more. The plots consistently show that the spectral power fluctuations in all cases clearly conformed to the line of maximum entropy on the time scale of a few seconds. How well the spectral-power fluctuations conformed to the maximum-entropy line is quantitatively summarized in Figure 5, showing that up to d = 3 sec the deviations were small (based on the actual range of values obtained from the individual participants rather than relying on summary statistics such as confidence intervals, p values, etc.). p_term is precisely defined in the context of the mathematical model. We suspect that the reviewer may be skeptical of the biological plausibility of p_term; if so, we respect their stance. We acknowledge in the manuscript that we provided a simplest mathematical model that captured the essence of the relevant dynamical features without a strong emphasis on biological plausibility.

The reviewer invite the authors to revisit this work that may have a potential impact with a more rigorous presentation and argumentation. The reviewer encourage such work with clear statements about synchrony, maximum entropy principle and stationarity in EEG recordings.

We thank the reviewer for many valuable comments. We have substantially revised the methods section based on the reviewer's helpful suggestions. However, we prefer to keep the general structure of the introduction, methods, and results sections, though it may be somewhat unconventional. As discussed above we believe that it is more effective to develop the theoretical and experimental results in tandem than splitting them across the three sections. It appears that the major concerns raised by the reviewer regarding “synchrony, maximum entropy principle, and stationarity” primarily reflect confusion/misunderstandings regarding the definitions of those terms. We hope that our point-by-point response above has resolved at least some of the misunderstandings. We have also revised the manuscript to improve conceptual clarity.

Our results are solid in terms of demonstrating that slow adjustments of average spectral power on the timescale of a few seconds while maintaining maximum entropy explains most of the characteristic U-shaped deviations of EEG spectral-power temporal distributions from stochastic (exponential) dynamics. We believe this finding makes a substantive scientific contribution. The inference that these slow adjustments are accomplished by slowly adjusting the probability of synchronization (p_term; 1–p_term to be precise) depends on the biological plausibility of the simple mathematical model we presented. Nevertheless, our results stipulate that any model should directly or indirectly include a parameter equivalent to $1/p_{term}$ that is directly related to the temporal average of the size of synchronized population (*Eq*. 5).
